# Moving beyond non-engagement on regulated needle-syringe exchange programs in Australian prisons

**DOI:** 10.1186/1477-7517-6-7

**Published:** 2009-05-04

**Authors:** Daniel Mogg, Michael Levy

**Affiliations:** 1 School of Psychology, Psychiatry and Psychological Medicine, Monash University, Melbourne, Victoria, Australia; 2 Victorian Institute of Forensic Medicine, Monash University, Melbourne, Victoria, Australia

## Abstract

**Background:**

Australia is at a fork in the road with the possibility of a needle-syringe exchange program (NSP) to be introduced at the new prison in the ACT. However, the current situation is characterised by non-engagement from major stakeholders. We explore why informed discussion will not be enough to convince prison officers, policy makers and the wider community of the benefits of prison-based NSPs. Other methods of engagement and communication will be proposed – in that may provide avenues for "breakthrough".

**Methods:**

A review of the literature on needle-syringe exchanges and harm reduction strategies within the context of prisons and prisoner health was conducted. Literature on strategies to change attitudes and move beyond intractable situations was also consulted. In addition, one author, DM, conducted a two-hour interview with an ex-prison officer.

**Results:**

No studies were found which investigated the potential efficacy of interventions to modify attitudes or behaviours in the specific context of introducing an NSP into a prison. Nonetheless, several theories were identified which may explain the failure of informed discussion alone to create change in this situation and may therefore lead to suggestions for engagement and communication to move towards a resolution

**Discussion:**

Cognitive-behavioural therapy highlights the importance of individual cognitions and how they shape behaviours in any change campaign. Social identity theory emphasizes changes to social processes that may open the prison officer workforce to change. Peace studies also suggests socialization strategies such as observing an established and effective prison-based needle-syringe exchange. Social marketing provides suggestions on how to sell an exchange to ensuring the benefits are framed to outweigh the costs.

**Conclusion:**

Psychology, peace studies and social marketing all agree people's views must be carefully collected and analysed if people are going to be convinced to consider and discuss the issue. By understanding the views and their underlying motivations of those who oppose NSPs, it will be far easier to influence these views. Furthermore, involving all stakeholders, especially prison authorities, will help create a sense of ownership of a solution and therefore increase the chances of that solution succeeding.

## Background

Australia is at a 'fork in the road' with the real possibility of a needle-syringe exchange program (NSP) being introduced at a new prison, the Alexander Maconochie Centre, in the Australian Capital Territory (ACT). The ACT Human Rights Commission has recommended that, " [a] pilot program for a needle and syringe exchange with provision for safe disposal of needles should be developed for the Alexander Maconochie Centre....." [1].

However, there is currently non-engagement between major parties and overt resistance from others on this issue. We examine the discourse between these stakeholders regarding the introduction of an exchange and explore why, despite the weight of health, human rights and economic arguments, there continues to be strong opposition from various sectors. We examine why informed discussion alone is not enough to move beyond non-engagement and convince prison officers, policy makers and the wider community of the benefits of prison-based injecting equipment exchanges. Finally, other methods of engagement and communication will be proposed, particularly from the fields of psychology, peace studies and social marketing, which may provide avenues for a resolution via effective consultation and collaboration.

Injecting drugs is a major risk factor for contracting bloodborne viruses (BBV) such as the human immunodeficiency virus (HIV) and the hepatitis C virus (HCV) [2]. Despite being a global issue, the response of governments differs from country to country. This difference is evident when examining the approaches to intravenous drug use in prisons. Prison-based NSPs exist in nine countries (Switzerland, Germany, Spain, Moldova, Kyrgyzstan, Belarus, Luxembourg, Armenia and Iran) with several more considering their introduction [3]. There have been several detailed evaluations of NSPs in these prisons [e.g., [2-4]], which have shown positive results including the reduction of equipment sharing; the reduction of HCV infections; and equipment not being used as weapons against staff. These findings have lead to a position being adopted by the World Health Organization (WHO) [4], supporting the introduction of prison-based NSPs.

In Australia, the Australian National Council on Drugs, the Commonwealth Government's principal advisory body on drugs policy, supports the trial of NSPs in prisons [5]. The 2002 Review of the National Hepatitis C Strategy recommended that Australia look towards implementing the "lessons learnt" from overseas applications of harm reduction strategies [6]. However, Australia's application of these lessons in harm reduction is mixed (see Table 1) [7]. Despite international evidence and policy supporting NSPs in prisons, no Australian jurisdiction currently allows them.

**Table 1:** Use of Harm Reduction Strategies in Australian States and Territories

**Jurisdiction**	**Bleach**	**Needle-Syringe Program**
New South Wales	Available anonymously	Not available

Northern Territory	"Cleaning Agents" available	Not available

Queensland	Not available	Not available

South Australia	Not available	Not available

Tasmania	Not available	Not available

Victoria	Available anonymously	Not available

Western Australia	"Cleaning Agents" available	Not available

Australian Capital Territory	"Cleaning Agents" available	Not available

[Adapted from [7]]

The absence of regulated NSPs in Australian prisons is alarming given the findings of the 2001 New South Wales Inmate Health Survey which found that 43% of females and 24% of males had injected drugs whilst in prison. Of those, 72% of females and 67% of males had reused the needle and syringe after someone else [8]. These findings support those of an earlier study across four major Australian cities (i.e., Melbourne, Sydney, Adelaide and Perth) which also found high rates of needle and syringe sharing amongst intravenous drug users in prisons [9]. In this context, approximately 40% of the Australian prison population have HCV and it is believed that the prevalence amongst the female prisoner population is closer to 65% [10].

### The ACT Situation

The ACT Legislative Assembly's Standing Committee on Health investigated the issue in 2003 and recommended that an NSP be trialled at the new prison. This was reiterated by the local Winnunga Nimmityjah Aboriginal Health Service which recommended the introduction of an NSP at the new prison in its report "You do the Crime, You do the Time" [11].

The ACT's Human Rights Commission investigated the operation of the current ACT correctional facilities in 2006 and 2007 to ascertain their compliance with the ACT's *Human Rights Act 2004*. Of the Commission's 96 recommendations, one was for the trial of an NSP. This recommendation was based on a prisoner's right to life, which includes protection from infectious diseases, as well as the right to the highest attainable standard of health [1]. Despite this endorsement, the ACT Government's actions have been far from supportive. In fact, their position has moved between 2005 and 2008, gradually distancing themselves from this intervention.

In November 2005, the then Health Minister of the ACT, Mr Simon Corbell MLA, spoke on local radio and advised that the Government was considering an NSP trial at the new prison. Mr Corbell stated, "what we want to do is stop the spread of disease that comes from sharing needles" [12]. However, by 28 August 2007, the position confirmed by the current Health Minister, Ms Katy Gallagher MLA, was that "...the Government has not supported the inclusion of a needle and syringe program at this stage" [13]. The Government has stated that if further consideration of a trial NSP is warranted, ACT Health will investigate the feasibility of introducing such a trial at the Alexander Maconochie Centre.

There have been reports of lobbying by the prison officers' union (Community and Public Sector Union) who admitted its members "are very strongly opposed to needles in prisons" [14]. A clause has even been inserted into the new workplace agreement for ACT Corrective Services, which states, " [w]ithout implying prior agreement, and for the safety of staff, no needle exchange program, however presented, shall be implemented without prior consultation and agreement by the parties to this Agreement on how such a program can be implemented" [15].

Opposition by correctional services officers is partly due to fears for their own physical safety, which were seemingly vindicated in Australia on 22 July 1990 when a New South Wales correctional officer, Mr Geoffrey Pearce, contracted HIV after being stabbed by a prisoner with a syringe that contained HIV-infected blood. Pearce died of AIDS in 1999 and prison officers believe such an incident would become more common if injecting equipment was introduced through an NSP [16].

Other arguments mounted to oppose the NSP include the apparently paradoxical message of sending people to prison for drug-related offences and then giving them injecting equipment to assist in their drug use [1]. Opponents also suggest that providing injecting equipment in prisons would actually encourage drug use [3]. Furthermore, it has been suspected by correctional staff that this increase in drug use could lead to more adverse events in prisons such as drug overdoses [1].

This impasse between evidence for NSPs in prisons and understandable, but unsubstantiated concerns over its risks, is echoed in the evidence for recent guidelines regarding hepatitis C prevention, treatment and care in Australian prisons [17]. Whilst acknowledging the complexity of the issue, the guidelines fail to follow their own evidence base and advocate for the trial of an NSP, instead providing suggestions to Australian states and territories *if *they chose to pursue that avenue [18].

In summary, a situation now exists where on one side there is evidence that support NSPs in prisons, and on the other there is fear and anxiety that staff members might be attacked or drug use will be inadvertently promoted. Fundamentally, neither side is able to convince the other of its arguments and instead of resolving the issue, there is a stalemate of non-engagement. Informed discussion is not enough to convince prison officers, policy makers and the wider community of the benefits of NSPs.

The aim of this study is to examine possible explanations for the impotence of informed discussion in this situation and to make suggestions for other methods of engagement and communication to move towards a resolution.

## Method

Evidence for this report was collected over a six-month period using the online databases PsycINFO and MEDLINE. The search terms used were needle-syringe exchange, needle-exchange program, harm reduction, prison, and prisoner health. Literature on strategies to change attitudes in general and move beyond intractable situations at the micro-level of individuals, meso-level of social groups, and macro-level of population groups were also consulted as well as methods of mass behaviour change that have received attention in recent times. Sources were limited to those in English and included Australian and international published reports, journal articles, conference presentations, government publications, and non-government organisation publications. Secondary sources were searched through the references of primary sources.

In addition, one author, DM, conducted a two-hour, unstructured interview with an ex-prison officer who was known to the authors. The interviewee consented to participate on the basis of anonymity and was not reimbursed for his time.

## Results

No studies were found which investigated the potential efficacy of micro, meso, or macro-level interventions to modify attitudes or behaviours in the specific context of introducing an NSP (or any other harm reduction measure) into a prison. Nonetheless, several theories were identified which may explain the failure of informed discussion alone to create change in this situation and may therefore lead to suggestions on other methods of engagement and communication to move towards a resolution.

At the level of the individual (micro), cognitive-behavioural therapy (CBT) suggests that it is not what actually happens to people which influences their feelings and behaviours, but rather individuals' *perceptions *of what happens [19,20]. A cognitive-behavioural therapist will examine an individual's thinking errors or "cognitive distortions" as a possible source of inability to cope adaptively and manage stress. Generally, when a psychologist encounters an individual with cognitive distortions they would use CBT to challenge these cognitions and help the person modify these thoughts into something more adaptable [20].

Social identity theory (SIT) assumes a group perspective and suggests that an important part of an individual's self-concept is how that individual defines themselves through the social groups to which they belong [21]. SIT, and the related self categorisation theory (SCT), propose that individuals will categorise themselves as part of a particular social identity to the extent that this identity is made salient in a given context [22]. These social identities give the individual a group prototype that prescribes beliefs, attitudes and behaviours for the individual as part of the group [23]. Research has begun on the possibility of modifying social identities to include particular attitudes or behaviours [e.g., [24]].

No research was found that has investigated the actual attitudes and behaviours that are associated with a prison officer's social identity. The interview with the ex-prison officer provided a small insight given the little research that is available. He explained that the role focussed on regulating behaviour, enforcing rules and maintaining the peace. Supporting the health of prisoners is viewed as the job of the "care bears" – the doctors, nurses and allied health staff. Training for new recruits includes discussion of human rights and health and safety however older prison officers, who did not receive this training, often fail to observe the values the training promotes and therefore nullify the new recruits' training by socialising them into old correctional attitudes and behaviours. Needles are feared and demonised as weapons and change is generally resisted. Prison officers "know best" when it comes to prisoners, and have to face a part of society that the rest of the community wants to forget about.

Peace studies examines the prevention, de-escalation and solving of conflicts [see [25]] and is therefore often applied to the macro-level of population groups. It suggests that conflict most often occurs when basic human needs, including those such as the need for physical safety and control, are "denied, threatened or frustrated" [[26], p. 29]. One technique posited by peace studies to move beyond intractable situations and create change is socialisation or learning attitudes, behaviours and norms through groups processes [26].

Social marketing is a behaviour change framework which uses marketing techniques to influence social and health behaviours with the goal of benefiting society. It is often applied at the group or population level through public health campaigns. Social marketing suggests that the likelihood of influencing behaviour is increased by a mutually beneficial exchange whereby the benefits of a particular product, service or behaviour appear to outweigh the costs, or, where the costs of not behaving in a particular fashion are perceived to outweigh the costs of acting [27].

To assist with this task, social marketers use the "four Ps". That is, the marketers consider:

▪ The **P**roduct – products are easier to sell when they are short-term, tangible, appealing, easy, trialable and carry low risks [27].

▪ The **P**rice – refers to the cost of the product in light of its benefits.

▪ The **Pl**ace – refers to how the product, or in this case, message is distributed to those who need to receive it.

▪ The **P**romotion – regards how the issue will be promoted in the broader context in which it exists.

## Discussion

Some theories (e.g., the Health Belief Model) propose that health attitudes and behaviours are based on reason and articulate constructs that rationally lead to particular behaviours. However, as had already been observed with attempts to introduce an NSP into Australian prisons, reason and evidence are not always sufficient to change attitudes and behaviours. Indeed, these types of theories have been criticised for assuming that people always act or think rationally and logically [28]. In many cases individuals or groups (e.g., trade unions) do not act rationally. Although objective evidence may support prison-based NSPs, prison authorities have concluded that introducing needles into prisons will increase the risk of injury to officers and promote drug use.

Notwithstanding the opposition to a prison-based NSP, there is mounting health, economic and human rights arguments for such change. Indeed, there are a number of strategies to both "push" and "pull" the issue forward. It is conceivable that eventually the ACT Government will be forced through the courts to introduce an NSP, such as when a case was listed before the New South Wales Supreme Court in 1996. A prisoner contended that the NSW correctional system was failing in its duty of care to protect him from sexually transmitted diseases by not supplying condoms. The case was never heard as the prisoner died during the proceedings but it is interesting to note that the prison authorities introduced condoms as a harm reduction strategy soon after [29,30].

In 2006, the case of Shelly v. the United Kingdom was brought before the European Court of Human Rights on the issue of a prisoner's right to access an NSP in a prison. The Court found in favour of the English Government but relied on the Margin of Appreciation [31]. This principle allows the court a certain amount of latitude in its decision in recognition of the court's jurisdiction over multiple states and the various ways one decision would be interpreted in each state [32]. A similar case could occur in the ACT where the Margin of Appreciation is irrelevant.

Although interesting, these "push" tactics are outside the scope of this discussion and so instead the focus will be on how to "pull" the relevant stakeholders around to the idea of trialling an NSP at the new prison.

Looking at the individual level, the opposition of correctional authorities to NSPs by avoiding all evidence for such could be framed as cognitive distortions. Table 2 illustrates types of thinking errors and how prison officers may be exhibiting those errors. For example, prison officers are using a mental filter whereby they are selectively attending to a single negative detail to the exclusion of other details when they highlight the case of Mr Geoff Pearce yet fail to recognise that he was attacked with a needle in a prison *without *a regulated NSP. Furthermore, there has been no significant response to this incident by prison authorities and so their staff members continue to operate under the same working conditions that existed when Mr Pearce was stabbed. Some would argue that it is mistaken to pathologise prison officers' genuine concerns for their safety. It is one thing to have concerns and it is another to avoid the issue altogether. In a human rights and public health framework, prison officers cannot ignore the risks to the health of prisoners or themselves.

**Table 2:** Possible Cognitive Distortions of Prison Officers regarding NSPs

**Distortion**		**In this situation...**
All-or-nothing	Seeing things in black and white (absolute) categories	"Prisons should aim to be drug-free or should just give up"

Over-generalisation	Seeing a single negative event as a never-ending pattern of defeat	"Because of what happened to Geoff Pearce, all needles are unsafe/infected"

Mental filter	Picking out a single negative detail and dwelling on it to the exclusion of other details	"It doesn't matter that Geoff Pearce was stabbed in a prison without a regulated NSP, what matters is that he was stabbed and later died".

Disqualifying the positive	Rejecting positive experiences by insisting they don't count	"Positive experiences in Europe don't count – things are different there"

Jumping to conclusions	Mind reading, fortune telling, etc.	"Trialling an NSP will promote drug use"

Minimisation	Shrinking the importance of things inappropriately	"It doesn't matter that we will be able to control previously uncontrolled contraband"

Emotional reasoning	Thinking that negative emotions reflect the way that things really are.	"The idea of a prison-based NSP makes me feel unsafe, therefore it must be unsafe".

Generally, CBT would be used with an individual experiencing cognitive distortions to challenge these cognitions and help the person modify these thoughts into something more adaptive [20]. However, in this case, the "distortion" is occurring at an institutional, rather than individual level, and correctional services officers are not distressed by their own cognitions. They are not seeking help and would almost certainly not consent to therapy to overcome these cognitions.

Adopting a social group perspective, the specific social identity of being a prison officer may also operate to stifle rational engagement with the issue. Prison officers' social identities are made relevant within their work setting by:

• a standard uniform for all members of the group;

• a specific location where the identity is elicited (i.e. the prison);

• organisational and industrial structures which bind the group members together;

• regular contact with a group the officers do not belong to (prisoners) that nevertheless reinforces the roles of the prison officer social identity;

• teamwork being encouraged given the potential risks to personal safety;

• longer-than-usual shifts at odd hours that reduce interaction with the rest of the community and reinforce interaction with other prison officers; and

• physical isolation from the community.

The sample of one ex-prison officer cannot be generalised to the broader ex- or current-prison officer population. Nonetheless, this interview provided an avenue, in a non-hostile context, to discuss and seek clarification on many issues identified through the literature review of one of the main, unmoveable stakeholder groups. It is suggestive of a mismatch between prison officers' attitudes and behaviours as elicited by their group identity (e.g., prisoners' health is someone else's concern), and the kind of attitudes and behaviours that would be amenable to a prison-based NSP (e.g., prisoners' health is part of my responsibilities). This situation is compounded by the cognitive distortions discussed earlier. Asking prison authorities to consider NSPs is expecting them to change their thinking and modify their social identities.

Unfortunately, SIT does not easily lend itself to practical interventions to modify specific attitudes and behaviours. Nevertheless, the theory could be used to encourage a more open social identity amongst prison officers that is more permeable to general community attitudes. The best way to achieve this is to make any changes as palatable as possible to prison officers, possibly by increasing career opportunities and reducing job dissatisfaction. Strategies could include rotating staff into and out of the new prison to ensure staff attitudes are invigorated through different exposures and employee diversity or enhancing staff training and professional development to professionalise the workforce and even encourage a sustainable and constructive level of staff turnover. Another approach could be to regularise prison officers' working hours to better fit with community standards so that the possibility of interacting with the general community is increased.

With respect to peace studies, prison officers' need for security and control is seemingly at odds with prisoners' need for security from BBVs. A resolution of this conflict is frustrated because opposition to NSPs is well organised and easily accessible to the public (e.g., ACT Corrective Services, ACT Department of Justice and Community Services, and the CPSU) whereas supporters of NSPs are less organised, lack widespread public credibility, work surreptitiously or are restrained by other political considerations.

Nonetheless, peace studies suggests socialisation as a technique to move beyond the seemingly intractable conflict between prisoners' and prison officers' needs. This approach may take a number of forms but one way is to facilitate prison officers' observation of NSPs that have been introduced in prisons and are working well, such as the prisons in Spain or Germany. This could allay concerns for personal safety and increase a sense of personal efficacy by seeing that such a program can be implemented. However, this approach would need to be carefully managed to ensure that other factors (e.g., motivation to sabotage the process, low identification with prison officers at the observed prison and the failure of observations to be appropriately diffused from the observers to their work colleagues) are minimised and do not compromise socialisation efforts.

The impetus that arises from the objective evidence is further undermined by other stakeholders who are also unmotivated to engage or even motivated in a different direction. It is likely the ACT Government did not want to appear weak on drugs, or on prisoners when an election was approaching (October 2008), particularly as it did not then, and continues not to enjoy bipartisanship on the issue. Furthermore, it is conceivable that the Government wants to appease the corrective services workforce which is currently in short supply with a growing demand. The Government's decisions to date go largely unchallenged because the broader community is not particularly interested in an issue that is too distant from their everyday lives.

Social marketing is a framework that examines motivations to undertake a particular action or subscribe to a particular belief by focussing attention on the costs or benefits to a particular target group. In this situation, the product that needs to be marketed is the concept that an NSP in the new ACT prison is actually a favourable outcome for all parties involved. So as to optimise the mutually beneficial exchange to influence behaviours, the costs and benefits of an NSP to major stakeholders needs to be considered. Table 3 illustrates the needs and possible costs and benefits of an introduction of an NSP for each group. For example, in the case of politicians, efforts must be made to emphasise that NSPs are a strong public health measure, would save money on treatment costs and increase human rights credibility. These must appear to outweigh the perceived costs and so politicians need to be shown how to achieve all this and not appear soft on drugs, soft on prisoners or unconcerned about prison officer safety.

**Table 3:** Analysis of Costs and Benefits of NSPs to Stakeholders

**STAKEHOLDER**	**NEEDS**	**BENEFITS FROM NSPs**	**COSTS FROM NSPs**	**POTENTIAL ROLE**
Prisoners	▪Access to clean syringes to prevent transmission of BBV ▪Confidentiality to reduce stigma/discrimination based on NSP usage	▪Clean syringes to reduce needle-sharing ▪Reduced risk of contracting BBV	▪Possible stigma/discrimination from correctional staff if program not correctly implemented.	▪Participate in NSPs by using clean needles

Correctional Officers	▪Feel safe ▪Feel in control of the prison ▪Maintain their role of being "tough on drugs"	▪Able to regulate an unregulated item	▪Feeling more at risk of needle injury including attack ▪Feeling soft on drugs	▪Help ACT Health develop NSP to accommodate their concerns

Politicians	▪Appear tough on drugs ▪Deliver on promise of prison based on human rights framework ▪Public confidence in new prison ▪Appear to maintain public health	▪Improve human rights "credibility" of prison ▪Reduce costs associated with treating/managing BBVs ▪Appear strong on public health and infection control	▪Appearing soft on drugs or even condoning their use ▪Appearing to be soft with prisoners ▪Backlash from correctional officers/union	▪Allow NSP to proceed ▪Provide resources for proper trial and evaluation

Community	▪Feel prisoners are suitably punished for crimes ▪Feel safe from threats to public health	▪Reduce risk of infection for family/friends of prisoners ▪Feeling there is strong public health intervention	▪Feeling prisoners are being indulged ▪Feeling tax-payers money is being wasted	▪Influence politicians and the media ▪Strengthen concerns for public health

Media	▪Commercial success ▪Good stories	▪Compelling stories (both political and human stories)		▪Set agenda ▪Inform public ▪Influence politicians

Applying the concept of the "four P's" to an NSP in the ACT prison, discussion should be about a trial of an exchange for a fixed-term (e.g. 18 months). The focus should be on increasing prison officer safety and regulating a currently unregulated system. The costs of an NSP are different for each stakeholder but the overall costs (i.e., prison officer resistance, set up costs and public concern) can be considered as fleeting when compared to the costs of not introducing an NSP (i.e. increasing levels of BBVs in prisons, increasing risk of these viruses spreading to the community, reputation as weak on public health and unconvincing commitment to human rights). It has been noted that whilst new recruits are a key group to educate about this issue, more experienced employees also need to be targeted. Given the hierarchical nature of the workforce it would be advantageous for this to unfold in a top-down manner where respect for rank will translate into respect for views. Promoting an NSP in the new prison will compete with negative messages and efforts must be made to counter these arguments and frame NSPs in prisons in a beneficial way. Table 4 below illustrates current messages that need to be reframed.

**Table 4:** Current Messages which need Reframing

**Current message...**	**Replace with...**
Prisoners don't deserve to be looked after like this	This is about protecting prison officers from unregulated NSPs

NSPs put prison officers at risk	Regulated NSPs address the risks that currently exist and were demonstrated in the case of Geoff Pearce

NSPs will increase drug use	NSPs will provide a useful avenue to engage with drug users and perhaps divert them into treatment.

An NSP is essentially condoning drug use	NSPs are about controlling injecting equipment and they currently exist in the community alongside bans on drugs.

Ultimately, the contributions that cognitive-behavioural theory, social identity theory, peace studies and social marketing could make to convince stakeholders to trial an NSP in a prison are untested. While the theories may be useful, particularly in understanding the resistance to prison-based NSPs and suggesting ways forward, their real value must be tested empirically. Research is required that will examine stakeholders' attitudes, beliefs and behaviours with respect to NSPs and test the utility of the theories mentioned to modify these.

## Conclusion

A policy environment exists both nationally and internationally that would support the introduction of an NSP at the Alexander Maconochie Centre. However, the situation in the ACT shows that there is a lack of political will to introduce such a program. This non-engagement between the major parties ranges from cautious resistance from the Government to overt defiance from prison officers. Focusing on prison officers, their opposition can be conceptualised from a cognitive-behavioural perspective as cognitive distortions whereby they fail to engage with the evidence that clearly challenges their position. It is also apparent that their non-engagement is fuelled by a well-maintained social identity that demonises needles and generally resists change.

Whilst psychology, peace studies and social marketing contribute different ideas to the issue there are two important points on which all agree. If people are going to be convinced to consider and discuss the issue, their views must be very carefully collected and analysed. By understanding the views and their underlying motivations of those who oppose NSPs, it will be far easier to influence these views. Furthermore, involving all stakeholders, especially prison authorities, will help create a sense of ownership of whatever solution is devised and therefore increase the chances of that solution succeeding.

Whilst these suggestions sound promising, further research is vital to test the applicability and effectiveness of these perspectives in this context. In addition, strong political leadership will be paramount in utilising these suggestions to move the parties from non-engagement to constructive discussion.

Ultimately, biology will prevail if policy fails. Prison authorities and the broader community must constructively confront the challenges posed by prison-based NSPs. Not to do so will lead to ongoing preventable breaches of health rights and human rights.

## Competing interests

The authors declare that they have no competing interests.

## Authors' contributions

DM conducted the literature review, interviewed the participant, contributed to its design and coordination and drafted the manuscript. ML conceived the initial study, assisted with the literature review, participated in the design and coordination and reviewed the manuscript. All authors read and approved the final manuscript.
